# Climate change aggravates bird mortality in pristine tropical forests

**DOI:** 10.1126/sciadv.adq8086

**Published:** 2025-01-29

**Authors:** Jared D. Wolfe, David A. Luther, Vitek Jirinec, Jeremy Collings, Erik I. Johnson, Robert O. Bierregaard, Philip C Stouffer

**Affiliations:** ^1^ College of Forest Resources and Environmental Science, Michigan Technological University, Houghton, MI 49931, USA.; ^2^ Biological Dynamics of Forest Fragments Project, Instituto Nacional de Pesquisas da Amazônia (INPA), Manaus 69060-001, Amazonas, Brazil.; ^3^ Biology Department, George Mason University, Fairfax, VA 22030, USA.; ^4^ Integral Ecology Research Center, Blue Lake, CA 95525, USA.; ^5^ Department of Biology, University of Oregon, Eugene, OR 97403, USA.; ^6^ Audubon Delta, National Audubon Society, New Orleans, LA 70119, USA.; ^7^ School of Renewable Natural Resources, Louisiana State University and LSU AgCenter, Baton Rouge, LA 70803, USA.; ^8^ Ornithology Department, The Academy of Natural Sciences of Drexel University, Philadelphia, PA 19103, USA.

## Abstract

Stable understory microclimates within undisturbed rainforests are often considered refugia against climate change. However, this assumption contrasts with emerging evidence of Neotropical bird population declines in intact rainforests. We assessed the vulnerability of resident rainforest birds to climatic variability, focusing on dry season severity characterized by hotter temperatures and reduced rainfall. Analyzing 4264 individual bird captures over 27 years, we found that harsher Amazonian dry seasons significantly reduced apparent survival for 24 of 29 species, with longer-lived species being more strongly affected. Our model predicted that a 1°C increase in average dry season temperature would reduce the mean apparent survival of the understory bird community by 63%. These findings directly link climate change to declining bird survival in the Amazon, challenging the notion that pristine rainforests can fully protect their biodiversity under increasingly severe climate conditions.

## Teaser

Climate change threatens Amazon rainforest birds, with harsher dry seasons significantly affecting their survival over 27 years.

## INTRODUCTION

Climate change presents a formidable challenge to biodiversity, particularly in extreme environments such as the Arctic, high-elevation regions, and deserts (*1*–*4*). In these systems, species are increasingly at risk of surpassing their thermal tolerances or straying outside their hygric niches (*5*, *6*). Previous studies have suggested that pristine lowland tropical rainforest, characterized as seamless expanses of forest untouched by industrial degradation, could serve as refugia against a changing climate (*7*, *8*). These areas provide stable, less seasonal weather, and continuous canopy cover, potentially protecting plants and animals from the harshest impacts of climate change (*7*–*10*). Alternatively, the stability of these forests may promote the evolution of long-lived species with little tolerance for environmental change, rendering them finely tuned and susceptible to small variations in understory conditions (*11*–*16*). Recent evidence supports this latter assertion, as wildlife populations within pristine tropical forests appear vulnerable to incremental shifts in climate, as evidenced by long-term declines in the physiological condition of tropical mammals (*17*, *18*) and abundances of tropical birds (*19*, *20*).

**Fig. 1.**
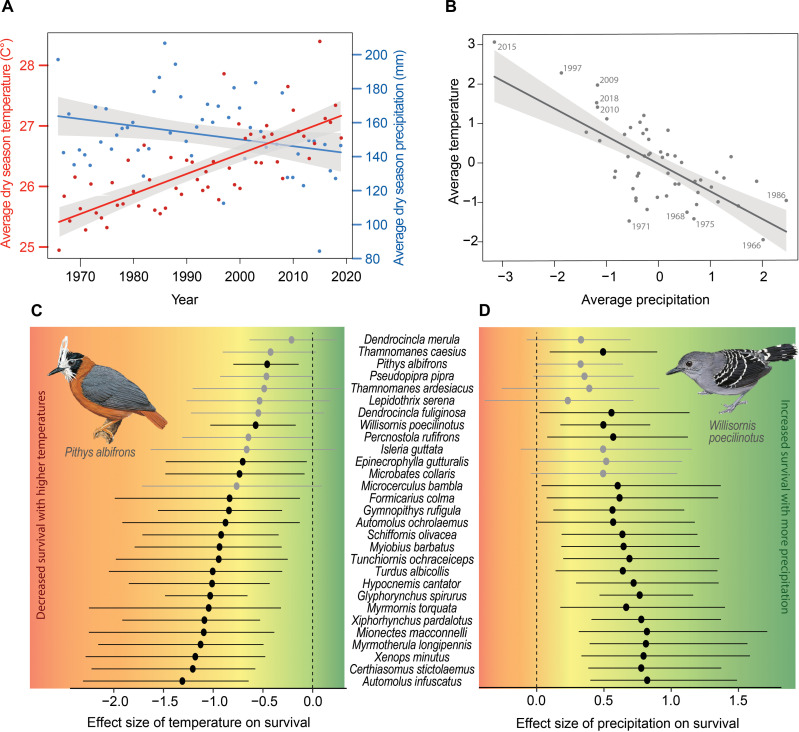
Dry season climate effects on apparent bird survival. Climate trends and survival impact on understory birds at the Biological Dynamics of Forest Fragments Project (BDFFP) near Manaus, Brazil. (**A**) Trends in average dry season temperature and precipitation (June to November) from ERA5 estimates for the BDFFP. Substantial increases in temperature and decreases in precipitation were predicted annually from 1966 to 2019 (see the Supplementary Materials for parameter estimates). (**B**) Correlation between standardized average dry season temperature and precipitation over time, indicating cooler, wetter conditions in the 1960s and 1970s, shifting to hotter, drier conditions in the 2000s and 2010s (see the Supplementary Materials for parameter estimates). (**C**) Beta estimates from bird species temperature survival models, with values left of zero indicating negative impacts of rising dry season temperatures on species survival. (**D**) Beta estimates from bird species precipitation survival models, with values right of zero indicating positive impacts of increased dry season precipitation on species survival; black beta estimates indicate no overlap with zero.

Research focused on the long-term demographics of tropical birds is rare. Three multidecadal and independent studies from Panama (*21*), Ecuador (*20*), and Brazil (*19*) are globally unique and provide insights into hemispheric-wide patterns of decline. In Panama, long-term monitoring revealed that 70% of 57 resident bird species declined in abundance over a 44-year sampling period (*21*). Among these declining species, 88% experienced losses of at least 50%. The researchers attributed these notable declines to factors such as habitat degradation and potentially climate change (*21*). This latter assertion was supported by an earlier study, which found that longer Panamanian dry seasons decreased the population growth rates of nearly one-third of the study species (*22*). In Ecuador, a 22-year study in pristine Amazonian forest showed similar trends, with overall capture and observation rates of birds dropping to approximately half of what were recorded during the first decade of the study, with insectivorous birds experiencing the steepest declines (*20*). In Brazil, over a span of more than 35 years in pristine Amazonian forest, approximately 50% of 79 species exhibited declines in abundance, with insectivorous birds experiencing the steepest declines (*19*). Results from Ecuador and Brazil indicate that even in the absence of direct anthropogenic landscape changes, insectivorous species are becoming rarer, with declines potentially driven by climate change. Recently, researchers have linked decreased rainfall and higher temperatures during the dry season (Fig. 1, A and B) to incremental decreases in body mass across an entire bird community in the Brazilian Amazon (*23*). These results underscore hidden biodiversity losses occurring in supposedly intact forests across the Neotropics.

Population declines stem from reductions in either survival or reproductive success, or both, with populations of longer-lived tropical birds expected to be particularly sensitive to changes in adult survival (*24*). Identifying how climate change affects these life history parameters is necessary to understand population-level vulnerability to climate change. As such, we hypothesize that declining populations of long-lived birds in seemingly pristine tropical rainforests result from a climatic impact, manifested through a process of understory drying. Our hypothesis is rooted in results from forest fragments (*25*), where hotter and drier microclimates diminish food resources (*19**,* *26*) and impose physiological stress on birds adapted to shaded forest conditions (*26*, *27*). If increasingly severe dry seasons are responsible for the declining abundance of central Amazonian birds within pristine forest, then we should detect a relationship between dry season severity and the apparent survival of understory birds.

## RESULTS

To investigate the hypothesis that severity of the dry season influences the apparent survival of understory birds in pristine rainforests, we used a hierarchical Bayesian Cormack-Jolly-Seber (CJS) model to analyze a globally unique bird capture-mark-recapture dataset gathered over a 27-year span from sites within pristine Amazonian forest at the Biological Dynamics of Forest Fragments Project (BDFFP). We estimated variation in the apparent survival of understory birds as a function of dry season severity, as measured by annual estimates of dry season temperature and precipitation. All told, we compiled 4264 annual captures of individual birds across 29 study species, from 20 sites, operated May through October, between 1985 and 2012.

Over 27 years, spatially explicit climate models from the ERA5 reanalysis (*28*) indicated that the average temperature during the dry season had increased by about 1°C while dry season rainfall decreased by about 10 mm (Fig. 1, A and B). We found that warmer dry season temperatures generally decreased annual apparent survival, with all 29 species showing negative median estimates for the effect of temperature (Fig. 1C). For 20 of 29 species, the 95% credible intervals associated with the temperature parameter did not overlap zero, indicating a significant negative effect. Even for the species with the weakest evidence, there was still an 84% probability that the effect of temperature was negative. Conversely, increased dry season precipitation generally increased annual apparent survival, with all 29 species showing positive median estimates for the effect of precipitation (Fig. 1D). For 21 of 29 species, the 95% credible intervals associated with the precipitation parameter did not overlap zero, indicating a significant positive effect. Even for the species with the weakest evidence, there was still a 77% probability that the effect of precipitation was positive. Overall, 24 of the 29 study species had 95% credible intervals that did not overlap zero for either temperature, precipitation, or both.

On the basis of the posterior distribution of mean climate effects, an increase in dry season temperature by 1°C diminished annual survival by an average of 63% while a decrease in dry season precipitation by 10 mm lowered overall annual survival by an average of 14%. For two commonly captured species, *Pithys albifrons* and *Willisornis poecilinotus*, we estimated survival to be 62% and 66% during the coolest year, and 21% and 16% during the hottest year, representing a 67% and 75% reduction in annual survival, respectively. Temperature and precipitation were strongly correlated (Fig. 1B), making their interactive effect on avian survival difficult to assess. Nonetheless, among the 27 study species where climate affected patterns of survival, average dry season temperature and precipitation explained on average 93% of the variance in annual survival.

**Fig. 2.**
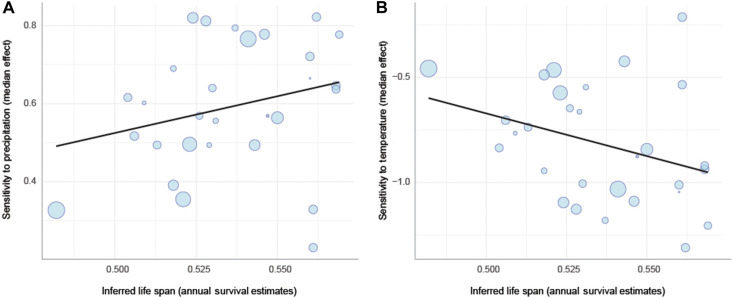
Life span and climatic sensitivity in Amazonian birds. Relationship between inferred life span and climatic sensitivity among understory birds at the BDFFP near Manaus, Brazil. (**A**) Sensitivity to precipitation plotted against inferred life span. Each data point represents a study species, with larger dots indicating more captures. The vertical positioning of dots reflects the median effect size of precipitation on survival, where higher positions suggest a greater positive effect of rain on apparent survival. (**B**) Sensitivity to temperature in a similar manner. Here, lower dot positions indicate a stronger negative impact of rising temperatures on survival, demonstrating that species with longer life spans tend to be more adversely affected by higher temperatures. These patterns illustrate the influence of life history variation on sensitivity to climatic shifts.

To test the hypothesis that longer-lived tropical birds are more sensitive to climatic variables than shorter-lived species, we conducted an additional analysis using the null survival model without climatic effects. This model generated baseline estimates of apparent annual survival for each species, where higher values indicated longer life spans. We then examined how these baseline survival estimates related to sensitivity to dry season severity using median values of temperature and precipitation from each species’ climate survival models as response variables. The regressions were weighted by number of captures to emphasize species with larger sample sizes. Our findings indicate that species with longer life spans, as suggested by higher survival rates, tended to be more adversely affected by higher temperatures (β = −0.039, SE = 0.01454, *P* = 0.01) and benefited from increased precipitation (β = 0.062, SE = 0.023, *P* = 0.01). Inferred life span accounted for approximately 21% of the variance in sensitivity to variations in dry season temperature and precipitation (Fig. 2).

## DISCUSSION

Our results provide the first evidence linking climate change, manifested through increasingly severe dry seasons, with decreased apparent survival estimates from a community of Amazonian birds. Over geologic and macro-evolutionary timescales, Amazonian bird communities have experienced average temperature changes exceeding 1°C. However, such changes likely did not occur as rapidly—over just two decades—as documented in this study, potentially impeding evolutionary adaptation to novel environmental conditions. The impact of climate change on the survival of understory birds likely reflects disruptions in the understory conditions that evolutionarily shaped their ecological niches and life history strategies (*11*–*14**,* *24*). Our findings support this perspective, where longer-lived understory birds appear more vulnerable to the worsening conditions of the dry season (Fig. 2). This observation underscores the complex relationship between life history strategy and climate change. Specifically, stable and biodiverse tropical environments fostered the evolution of a latitudinal gradient in life history variation (*15*). In this gradient, tropical songbirds prioritize adult survival and longer life spans with relatively lower reproductive output, whereas temperate songbirds typically exhibit shorter life spans coupled with higher annual reproductive efforts (*29*–*31*). This evolutionary adaptation to regional conditions may have rendered understory tropical birds particularly vulnerable to climatic shifts that adversely affect adult survival. These findings are especially alarming because they reflect demographic patterns of tropical birds within pristine rainforest, a biome thought to be resilient to the adverse effects of climate change (*8*–*10*). The acute effects of temperature on avian survival may help clarify previously documented reductions in understory bird populations (*19*) and associated changes in their behavior observed during intense dry season conditions (*26*).

Rapid increases in average temperature and decreases in average rainfall can affect birds in numerous ways, but are likely to affect insectivorous species—those most vulnerable to climate change—in two primary aspects. First, understory tropical birds are adapted to relatively cool and stable conditions where elevated temperatures may result in heightened physiological stress, leading these birds to select microclimate refugia, if available, to mitigate the deleterious impacts of understory drying. Recent studies from the Brazilian Amazon support this latter assertion whereby Jirinec *et al.* (*26*) observed variations in microclimates across seasons and elevations, with the driest conditions occurring at higher elevations and milder conditions in valleys. They found that *Formicarius analis*, an understory insectivorous bird, abandoned higher and more open locations and moved to valleys and areas with more cover, putative microclimate refugia, when subjected to hot and dry conditions. More recently, Jirinec (*27*) used temperature loggers attached to multiple understory insectivorous bird species to measure thermoregulatory behaviors and found that many species relied on bathing and rainfall to lower their internal body temperatures.

Conversely, studies from Panama did not find statistically significant associations between thermal physiological traits and temperature variation across various bird species that occupy dissimilar habitats and forest strata (*32*). Specifically, none of the four thermal physiological traits measured from wild birds that were captured and brought into a laboratory—lower critical temperature, upper critical temperature, thermal neutral zone breadth, and heat tolerance limit—showed a clear relationship with habitat type or vertical stratum. Unexpectedly, species from open habitats and the forest canopy exhibited narrower thermal safety margins compared to those from forested habitats and the forest understory (*32*). This contradicted predictions from previous studies that have shown strong links between local-scale temperature variation and thermal physiology and did not support the hypothesis that tropical understory birds are more sensitive to environmental change due to their constrained thermal physiology (*33*). Contradictory results from field observations in Brazil and laboratory measurements in Panama may reflect that laboratory conditions do not fully capture the stress that increased temperatures pose to birds when they are actively foraging and moving through their environment.

The second way hot and dry conditions can affect insectivorous species is by decreasing arthropod diversity and availability. In the tropics, arthropod assemblages vary as a function of microclimates where humid forests coupled with increased leaf area tend to host more abundant and diverse arthropod communities (*34*). This pattern reflects the physiological limitations of arthropods, small ectotherms with a high surface area–to–volume ratio, which face inherent risks of desiccation in arid microclimates. The risk of desiccation serves as an environmental filter, resulting in larger-bodied, less diverse, and less abundant arthropod assemblages in warmer and drier microclimates (*35*). For example, in the cloud forests of the Andes in northern Peru, researchers found that variation in rainfall resulted in a twofold change in arthropod biomass, with differences observed before and after the dry season (*36*). These results highlight the importance of climate fluctuations in shaping arthropod communities and suggest a link between increasing temperatures, decreased rainfall, and diminished food resources for birds in tropical forests.

Irrespective of whether hot and dry conditions lower apparent survival of birds through physiological stress, decreased food resources, or a mixture of both, climate change appears to affect birds during seasonally dry periods. These processes likely reflect the patterns of understory drying found in forest fragments and locations in pristine forest most susceptible to desiccation: upland (terra firme) forests, which are the focus of this study and represent roughly 82% of the central Amazon (*37*). Forests near streams or rivers may be more resilient to understory drying and subsequent loss of biodiversity (*26*). For example, at Cocha Cashu Biological Station in the Peruvian Amazon, a forest located within a mature flood plain, there has been little change in dry season temperature or precipitation (*38*), and bird community structure showed little change in two samples taken 36 years apart (*39*). The varied impacts of climate change on tropical bird communities and their habitats highlight distinct characteristics that make certain birds and their forests susceptible to a changing climate, whereas others remain resilient. Understanding the mechanisms through which climate change erodes biodiversity in pristine forests represents a pressing line of inquiry. Identifying the landscape characteristics that confer resilience to tropical forests and formulating policies to safeguard these resilient forests are essential steps toward ensuring the persistence of vulnerable tropical bird communities into the 22nd century.

## MATERIALS AND METHODS

### Field methods and study area

The study was conducted in an upland (terra firme) forest at the BDFFP, about 80 km north of Manaus, Brazil (*40**,* *41*). For this analysis, we used bird capture data from 20 banding transects within the KM41 control plot (pristine forest). Use of the terms pristine, intact, primary, and continuous tropical rainforest is nuanced and varies across the literature (*42**,* *43*). Here, we define “pristine forest” as a seamless expanse greater than 1000 km^2^ and free from remotely detected signs of human activity. Each bird capture transect hosted a line of 16 mist-nets (NEBBA-type ATX, 36 mm mesh, 12 × 2.5 m), where the bottom trammel of each net was set at ground level and opened from 0600 to 1400 for a single day of sampling. All captured birds were banded with uniquely numbered aluminum bands. We mistnetted birds between May through October, from 1985 to 2012.

### Statistical analysis

We used the EU Copernicus ERA5 climate reanalysis dataset (*44*), matching the spatial and temporal extent in Jirinec *et al.* (*26*), to generate annual estimates of temperature and precipitation averaged across dry season months, ranging from June to November. The ERA5 dataset combines climate modeling with historical data from weather stations and remote sensing, offering a spatial resolution of 0.25° latitude by 0.25° longitude and a temporal resolution of 1 hour (*28*). We extracted “2 m temperature,” which represents the air temperature estimated at 2 m above ground level, and “Total precipitation,” the aggregate of estimated precipitation over a given time period. For each year within our designated dry season time frame, we downloaded and averaged the daily mean temperature and daily total precipitation values. These averages were derived from four raster cells of the ERA5 grid, corresponding to the geographical area of our study, thus ensuring a localized and accurate representation of climatic conditions. These data provided us with a detailed annual climatic profile for each annual dry season from our study period, enabling us to assess the impacts of dry season climate variability on bird survival.

To estimate the global and species-specific effects of temperature and precipitation on bird survival, we fit a set of Bayesian hierarchical CJS where, for some models, the survival parameter was a linear function of either *z*-transformed temperature or *z*-transformed precipitation values, linked via a logit function. Following Yackulic *et al.* (*45*), we estimated the annual detection probability (*P*) and survival rate (φ) by tracking the marginal likelihoods of each bird’s capture history. For any year where sampling did not occur, we fixed *P* to equal zero (*46*). We removed all known juvenile birds from the dataset to reduce the influence of transient birds on model estimates. We developed a list of candidate models that included all possible combinations of time-dependent and time-independent *P*’s and φ’s (without the inclusion of climate variables; i.e., intercept-only models). We also fit models with either temperature- or precipitation-dependent φ’s and either time-dependent or time-independent *P*’s. Collinearity between temperature and precipitation (Pearson correlation = −0.86) prevented us from robustly testing additive and interactive effects between these climate variables; thus, we report these effects as potential alternative models of bird survival (though we recognize that precipitation and temperature are likely both synergistically affecting bird survival). For all models, we estimated species-specific *P*’s and φ’s. Intercepts and climate effect parameters, which made up the linear predictor for calculating φ, for each species were drawn from a shared distribution (partial pooling). This allowed us to additionally estimate global effects of temperature and precipitation on apparent survival. Conversely, each species’ *P* was estimated independently (no pooling). We used uninformative, uniform priors bound from zero to one to sample any given year’s *P*. Similarly, we used uninformative improper priors to sample global means and SDs of the intercepts and climate effects. Last, because we used the uncentered parameterization for fitting hierarchical models, we used standard normal priors to sample species-level deviations from the global mean intercepts and climate effects.

We fit all models using Hamiltonian Monte Carlo Markov chains in Stan (*47*) using the package rStan (*48*) version 2.21.2 in R (*49*) version 4.0.2. To evaluate whether the inclusion of temperature or precipitation within our CJS models was warranted, we used model selection among all the candidate models. We compared the predictive accuracy of each model by performing pareto-smoothed approximate leave-one-out cross-validation using the loo package (*50**,* *51*) in program R. Temperature or precipitation was considered an important explanatory variable if the expected log pointwise predictive density for its respective model was greater than those of each of the null models (table S1). To estimate the percent variation explained by our climate variables, we followed the ANODEV function in Program MARK by computing $\frac{-2\text{ln}\left[L\left(\varphi .P.\right)\right]+2\text{ln}\left[L(\varphi \text{CLIMATE}P.)\right]}{-2\text{ln}\left[L\left(\varphi .P.\right)\right]+2\text{ln}\left[L(\varphi tP.)\right]}$ for each iteration and reported the median of this distribution (*46*). The full list of species’ estimates is reported in table S2. We also provide baseline survival and recapture probability estimates and credible intervals from the fully constrained model [*P*(.) φ(.)] for the life-span analysis (table S3). We also provide an estimated global effect of precipitation and temperature from hierarchical models (fig. S1).

To examine the relationship between avian life span and climatic sensitivity, we conducted a weighted linear regression analysis. We used baseline estimates of apparent annual survival for each species as predictors, which were derived from a null survival model devoid of climatic effects (table S3). These estimates were used to infer the relative life span of each species, assuming that higher survival rates correspond to longer life spans. Median values of temperature and precipitation impacts, extracted from the posterior distributions of each species’ climate survival models, served as response variables (table S2). We accounted for variability in data reliability by weighting the regression analyses with the number of captures per species, thereby prioritizing species with more extensive capture data (table S2). The analyses were performed using the lm() function in R (*47*), version 4.0.2, with the weights parameter adjusted to reflect capture frequency.
