# Supplementary material for: Climate change aggravates bird mortality in pristine tropical forests
Source: Sci Adv. 2025 Jan 29;11(5):eadq8086. doi: 10.1126/sciadv.adq8086 (PMC11777245; doi:10.1126/sciadv.adq8086)

Science Advances
AAAS

Supplementary Materials for

Climate change aggravates bird mortality in pristine tropical forests

Jared D. Wolfe *et al.*

Corresponding author: Jared D. Wolfe, jdwolfe@mtu.edu

Sci. Adv. **11**, eadq8086 (2025)
DOI: 10.1126/sciadv.adq8086

This PDF file includes:

Supplementary Materials
Tables S1 to S3
Fig. S1

Supplementary Materials

In Figure 1, Panels A and B, we used linear regression in program R to examine correlations between average dry season temperature, measured in degrees Celsius, and year for the period 1966 to 2019 (β=0.033, SE=0.004, F-value=69.82, p-value<0.001). A similar regression analysis was conducted for average dry season precipitation, measured in millimeters, against year for the same period (β=-0.401, SE=0.184, F-value=4.78, p-value=0.03). Additionally, a regression analysis was performed to examine the relationship between dry season temperature and precipitation from 1966 to 2019 to assess their collinearity (β=-22.2, SE=3.1, F-value=50.0, p-value<0.001). All data and code are available via a GitHub repository (https://github.com/jeremyacollings/BDFFP_survival)

Table S1. Model comparison results using Pareto-smoothed approximate leave-one-out cross-validation (PSIS-LOO) to evaluate the predictive accuracy of eight candidate models for estimating bird survival. The ΔELPD (difference in Expected Log Pointwise Predictive Density) represents the decreased performance of each model relative to the best-performing model (φ(Temperature)p(.)). Standard deviations of ELPD are also provided to indicate the uncertainty in the model performance estimates.

Model	ΔELPD	SD ELPD
φ(Temperature)p(.)	0.0	0.0
φ(Precipitation)p(.)	-9.8	5.1
φ(.)p(.)	-561.7	10.7
φ(t)p(.)	-1486.4	22.1
φ(Precipitation)p(t)	-1913.0	28.1
φ(Temperature)p(t)	-1926.8	28.5
φ(.)p(t)	-2565.3	31.3
φ(t)p(t)	-4006.9	41.1

Table S2. Median estimates and 95% credible intervals for the effect sizes of average dry season precipitation and temperature on the apparent survival of 29 understory bird species in the Amazon rainforest. Negative median temperature effects indicate a decrease in annual survival with increasing dry season temperatures, while positive median precipitation effects indicate an increase in annual survival with higher dry season precipitation.

Species	Total captures	Median Temperature Effect	Lower Temperature Effect	Upper Temperature Effect	Median Precipitation Effect	Lower Precipitation Effect	Upper Precipitation Effect
Automolus infuscatus	112	-1.310	-2.310	-0.644	0.822	0.399	1.489
Automolus ochrolaemus	29	-0.876	-1.914	-0.130	0.569	0.007	1.176
Deconychura stictolaemus	88	-1.205	-2.223	-0.577	0.777	0.384	1.372
Dendrocincla fuliginosa	50	-0.546	-1.224	0.114	0.556	0.022	1.136
Dendrocincla merula	119	-0.212	-0.635	0.246	0.329	-0.075	0.697
Dixiphia pipra	372	-0.464	-0.930	0.005	0.355	-0.025	0.721
Epinecrophylla gutturalis	117	-0.703	-1.477	-0.059	0.517	-0.007	1.081
Formicarius colma	102	-0.835	-1.990	-0.128	0.616	0.075	1.349
Glyphorynchus spirurus	428	-1.031	-1.484	-0.655	0.766	0.467	1.163
Gymnopithys rufigula	232	-0.842	-1.556	-0.307	0.564	0.124	1.097
Hypocnemis cantator	112	-1.011	-1.848	-0.433	0.721	0.293	1.353
Isleria guttata	43	-0.663	-1.629	0.239	0.494	-0.119	1.154
Lepidothrix serena	111	-0.534	-1.269	0.177	0.231	-0.387	0.719
Microbates collaris	101	-0.736	-1.481	-0.081	0.494	-0.044	1.045
Microcerculus bambla	34	-0.765	-1.716	0.092	0.602	0.036	1.368
Mionectes macconnelli	187	-1.095	-2.250	-0.386	0.820	0.313	1.714
Myiobius barbatus	114	-0.937	-1.790	-0.311	0.646	0.183	1.213
Myrmornis torquata	28	-1.045	-2.249	-0.318	0.665	0.175	1.400
Myrmotherula longipennis	161	-1.127	-2.156	-0.494	0.812	0.394	1.567
Percnostola rufifrons	81	-0.646	-1.313	0.005	0.569	0.078	1.124
Pithys albifrons	476	-0.457	-0.798	-0.140	0.327	-0.012	0.641
Schiffornis turdina	101	-0.920	-1.712	-0.342	0.637	0.185	1.195
Thamnomanes ardesiacus	187	-0.423	-0.905	0.035	0.494	0.097	0.896
Thamnomanes caesius	168	-0.487	-1.207	0.289	0.391	-0.260	0.912
Tunchiornis ochraceiceps	57	-0.944	-1.979	-0.250	0.690	0.198	1.357
Turdus albicollis	91	-1.005	-2.049	-0.307	0.640	0.141	1.344
Willisornis poecilinotus	337	-0.573	-1.029	-0.170	0.496	0.176	0.844
Xenops minutus	64	-1.180	-2.283	-0.477	0.794	0.332	1.586
Xiphorhynchus pardalotus	162	-1.089	-1.914	-0.528	0.778	0.407	1.370

Table S3. Baseline survival (φ) and recapture probability (p) estimates along with their 95% credible intervals for 29 understory bird species in the Amazon rainforest. These estimates are derived from the fully constrained model (φ(.) p(.)), providing a comparative basis for understanding species-specific survival and detectability under non-climatic conditions.

species	phi median	phi low 95% CI	phi upper	p median	p low 95%	p upper 95% CI
Automolus infuscatus	0.562	0.485	0.668	0.343	0.221	0.498
Automolus ochrolaemus	0.547	0.440	0.667	0.376	0.166	0.654
Deconychura stictolaemus	0.569	0.491	0.680	0.353	0.216	0.523
Dendrocincla fuliginosa	0.531	0.397	0.646	0.211	0.089	0.456
Dendrocincla merula	0.561	0.485	0.665	0.358	0.234	0.510
Dixiphia pipra	0.521	0.418	0.606	0.123	0.076	0.196
Epinecrophylla gutturalis	0.506	0.348	0.595	0.207	0.101	0.409
Formicarius colma	0.504	0.307	0.601	0.123	0.051	0.321
Glyphorynchus spirurus	0.541	0.475	0.613	0.247	0.182	0.326
Gymnopithys rufigula	0.550	0.464	0.655	0.158	0.096	0.249
Hypocnemis cantator	0.560	0.477	0.685	0.160	0.085	0.279
Isleria guttata	0.529	0.398	0.637	0.246	0.103	0.518
Lepidothrix serena	0.561	0.482	0.691	0.185	0.103	0.305
Microbates collaris	0.513	0.369	0.605	0.213	0.107	0.414
Microcerculus bambla	0.509	0.321	0.605	0.341	0.122	0.830
Mionectes macconnelli	0.524	0.383	0.626	0.047	0.019	0.107
Myiobius barbatus	0.568	0.482	0.709	0.106	0.050	0.202
Myrmornis torquata	0.560	0.466	0.699	0.197	0.073	0.413
Myrmotherula longipennis	0.528	0.420	0.617	0.177	0.101	0.300
Percnostola rufifrons	0.526	0.383	0.625	0.199	0.096	0.389
Pithys albifrons	0.482	0.380	0.560	0.277	0.195	0.401
Schiffornis turdina	0.568	0.489	0.684	0.243	0.144	0.385
Thamnomanes ardesiacus	0.543	0.447	0.641	0.184	0.112	0.290
Thamnomanes caesius	0.518	0.373	0.626	0.095	0.044	0.195
Tunchiornis ochraceiceps	0.518	0.354	0.618	0.157	0.055	0.395
Turdus albicollis	0.530	0.401	0.632	0.189	0.089	0.363
Willisornis poecilinotus	0.523	0.432	0.595	0.236	0.166	0.334
Xenops minutus	0.537	0.416	0.654	0.163	0.070	0.333
Xiphorhynchus pardalotus	0.546	0.465	0.644	0.259	0.166	0.384

Figure S1. **Global Effects of Temperature and Precipitation on Bird Survival**. Median estimates and 95% credible intervals for the global effects of average dry season precipitation and temperature on the apparent survival of 29 understory bird species in the Amazon rainforest. Points represent the median effect sizes, while error bars indicate the 95% credible intervals. Negative effect sizes for temperature reflect a decrease in annual survival with increasing dry season temperatures, and positive effect sizes for precipitation indicate an increase in annual survival with higher dry season precipitation.

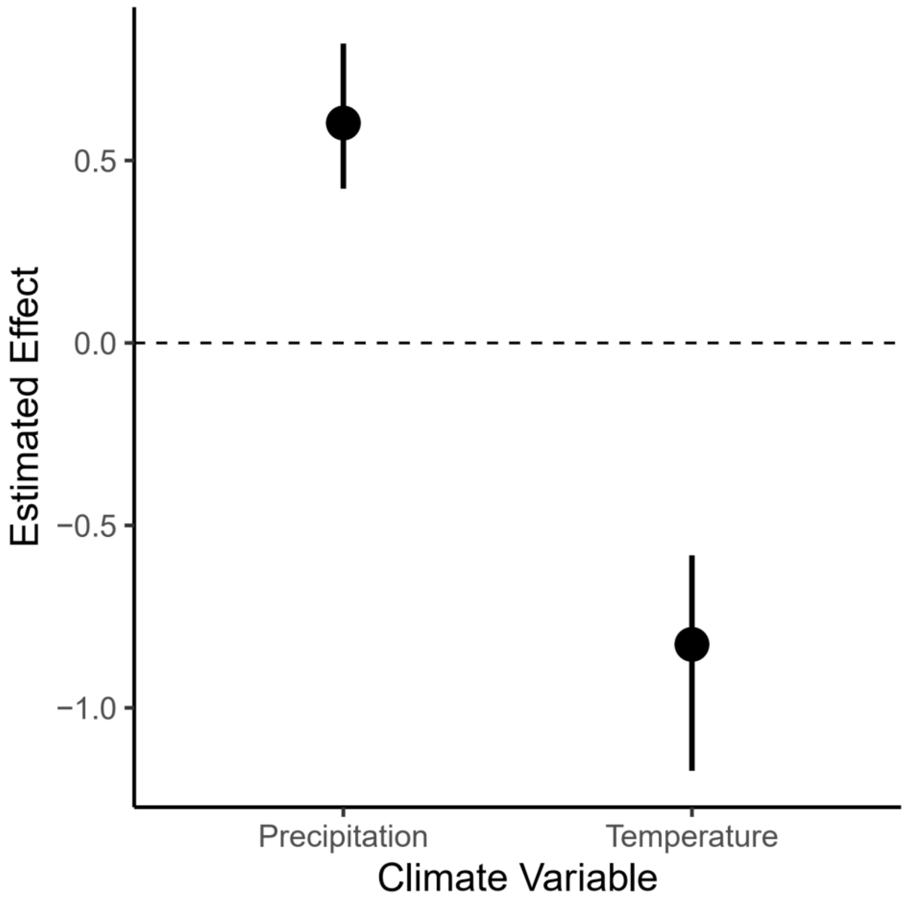

Supplement: Supplementary file 1 — Supplementary Materials Tables S1 to S3 Fig. S1 [file sciadv.adq8086_sm.pdf]
